# Recent Advances in Development of Small Molecules to Fight Cancer—Second Edition

**DOI:** 10.3390/molecules31050808

**Published:** 2026-02-28

**Authors:** Giulia Bononi, Carlotta Granchi

**Affiliations:** Department of Pharmacy, University of Pisa, 56126 Pisa, Italy

Cancer drug discovery continues to rely on the development of small molecules able to modulate key biological processes involved in tumor initiation and progression. Derived from natural products, endogenous metabolites or rationally designed synthetic scaffolds, these compounds can be exploited not only for therapeutic intervention but also for diagnostic applications. The contributions collected in this second edition of the Special Issue “Recent Advances in Development of Small Molecules to Fight Cancer” reflect this integrated approach, covering innovative chemical entities and strategies aimed at targeting cancer metabolism, signaling pathways, immune modulation and tumor aggressiveness.

Within this framework, significant advances in the fight against cancer have been driven not only by the development of new therapeutic agents, but also by the identification of novel biomarkers and molecular targets that are essential for guiding more selective and effective small molecule-based therapeutics. The first contribution of this Special Issue addresses the urgent need for improved therapeutic strategies for classical Hodgkin lymphoma (cHL), a B-cell malignancy characterized by frequent relapse despite effective chemotherapy [1]. By analyzing microarray gene expression data from cHL patients, the author identified a set of differentially expressed genes associated with disease progression. Functional and network analyses highlighted matrix metallopeptidase 12 (MMP12) and the chemokine ligand CCL22 as key upregulated genes involved not only in cHL progression but also in cancer-related pathways, including inflammatory signaling and metabolic regulation. Based on these findings, MMP12 was selected as a promising molecular target, and in silico docking and molecular dynamics simulations revealed that known anticancer compounds can stably bind to its active site. Overall, this study combines bioinformatics and computational chemistry approaches to propose MMP12 as a potential therapeutic target in cHL and to support the repositioning of small molecules as candidate inhibitors counteracting cHL progression.

Natural products remain a crucial source of inspiration for anticancer drug discovery, offering structurally diverse scaffolds with unique biological activities. Günther et al. investigated a series of oleanolic acid dimers (OADs) designed to enhance the anticancer potential of the parent natural triterpenoid oleanolic acid [2]. Fourteen dimeric derivatives were synthesized and evaluated across a broad panel of tumor cell lines, revealing a marked improvement in antiproliferative activity compared to oleanolic acid, with several compounds displaying IC_50_ values in the low micromolar range. Molecular docking studies supported a possible interaction with cancer-related protein targets, such as Focal Adhesion Kinase (FAK), highlighting the relevance of linker structure present in OADs for modulating their biological activity. In parallel, the dimers exhibited significant antioxidant properties and favorable predicted pharmacokinetic and ADMET profiles, suggesting that structural dimerization of oleanolic acid represents a promising strategy for the development of small molecules with anticancer potential.

Building directly on their previous investigation of OADs, the same research group further explored the chemical optimization of this triterpenoid scaffold through a straightforward acetylation strategy [3]. A new series of OADs was generated and structurally characterized, followed by an in silico structure–activity relationship (SAR) analysis to predict their pharmacological potential. Biological evaluation across four different cancer cell lines revealed a pronounced cytotoxic effect, often in the low micromolar range, accompanied by a favorable selectivity profile toward healthy cells. In addition, the acetylated derivatives retained notable antioxidant properties. Together, these results highlight how simple chemical modifications of natural product-based dimers can substantially enhance anticancer activity while preserving biological features.

Beyond isolated natural products and their derivatives, complex natural mixtures also represent a valuable source of bioactive small molecules with anticancer potential. Addressing the urgent need for effective therapies against glioblastoma multiforme (GBM), Chahla and colleagues investigated the anticancer potential of bee venom derived from *Apis mellifera syriaca* as a natural source of bioactive small molecules [4]. Through a combination of in vitro and in vivo approaches, the authors demonstrated a pronounced cytotoxic effect of bee venom on U87 GBM cells, accompanied by disruption of membrane integrity and activation of cell death-related pathways. Importantly, the antitumor activity observed in cell-based assays was corroborated in an animal model, in which bee venom treatment significantly reduced tumor burden and increased apoptosis. These findings support *Apis mellifera syriaca* venom as a promising and still underexplored reservoir of anticancer agents and provide mechanistic insights into their potential application against highly aggressive brain tumors.

Ma and coworkers explored inosine, a naturally occurring endogenous metabolite of adenosine, as an immunomodulatory small molecule capable of suppressing colorectal cancer (CRC) progression [5]. Moving beyond its known effects on T cells, the authors demonstrated that inosine reshapes the tumor microenvironment by increasing M1 macrophage markers CD86 and iNOS, thereby promoting macrophage polarization toward an antitumor M1 phenotype. Through both in vitro and in vivo models, inosine was shown to inhibit cancer cells proliferation and migration, reduce tumor growth and decrease proliferation markers, while enhancing macrophage-mediated antitumor activity. These findings highlight the therapeutic relevance of targeting immune cell plasticity using naturally occurring metabolites as an alternative and complementary strategy in cancer treatment.

Continuing in the field of naturally derived compounds, Zhang et al. investigated perillaldehyde, a monoterpene derived from *Perilla* species, as a potential small molecule agent against chronic myeloid leukemia (CML), a malignancy driven by the BCR-ABL fusion oncoprotein [6]. The compound was shown to disrupt mitochondrial function, induce reactive oxygen species and modulate apoptosis- and autophagy-related pathways in leukemic cells. Mechanistically, perillaldehyde targeted Heat Shock Protein 70 kDa (HSP70), a protein that plays a crucial role in the onset of cancer, leading to inactivation of BCR-ABL phosphorylation and suppression of downstream signaling, ultimately inhibiting cancer cell survival. These findings position perillaldehyde as a promising lead for developing novel CML therapeutics, highlighting the continuing relevance of natural products in oncology research.

Shifting the focus from naturally occurring compounds to rationally designed small molecules, Fortin and collaborators explored DAB-2-28, a *para*-aminobenzoic acid derivative, for its capacity to counteract epithelial–mesenchymal transition (EMT), a process linked to cancer invasiveness and metastasis, in breast cancer models [7]. Using both luminal (MCF-7) and triple-negative (MDA-MB-231) cell lines, the authors demonstrate that DAB-2-28 reduces key hallmarks of EMT, including cell migration, invasion and matrix metalloproteinase 9 protein (MMP9) activity, while inhibiting the phosphorylation of transcription factors involved in pro-EMT signaling pathways. These results indicate that EMT suppression is a central mechanism underlying the anti-tumor activity of DAB-2-28, highlighting the therapeutic potential of small molecules targeting macrophage-mediated inflammatory signals in breast cancer.

Rapidly proliferating cancer cells exhibit elevated glucose uptake, a hallmark exploited by emerging therapeutic strategies. In this context, the development of metal-based glycoconjugates has gained increasing attention as an effective approach to selectively target tumor cells. Brescia et al. authored a review summarizing recent progress in the design and application of these compounds for medicinal purposes [8]. The authors highlighted strategies that exploit the enhanced glucose uptake of rapidly proliferating cancer cells, allowing selective delivery of metal-based therapeutics and imaging agents. Beyond oncology, these conjugates are also being investigated for antimicrobial, antiviral, antiparasitic and neuroprotective applications. The review critically discusses advances since their previous 2015 article, exemplifying how the integration of metal complexes with sugar-based targeting moieties can expand the versatility and specificity of small molecule therapeutics.

Alongside therapeutic approaches, the development of efficient diagnostic tools is equally crucial in the fight against cancer. Tumor imaging currently relies on radioactive tracers such as fluorodeoxyglucose (FDG), but their limited half-life and high costs restrict widespread use. In this regard, metal-based glycoconjugates can serve as versatile tools for imaging applications. Bononi et al. developed a glucose-conjugated cyclopentadienylrhenium(I) tricarbonyl (Cp[Re(CO)_3_]) probe as a non-radioactive alternative for infrared (IR)-based detection of cancer cells [9]. By exploiting the cellular transparency window between 1800 and 2200 cm^−1^, the probe enables selective IR visualization of cancer cells. This strategy provides a promising approach for non-invasive tissue imaging and highlights how metal-based glycoconjugates can expand the utility of small molecules beyond therapeutic applications, opening new avenues for diagnostic purposes.

Collectively, these contributions illustrate the multifaceted strategies currently being pursued to modulate tumor growth, metabolism, immune response and diagnostic imaging, underscoring the continued importance of small-molecule research in advancing cancer therapy and diagnosis.
